# Supplementary figures and images for: The role of RNA in the maintenance of chromatin domains as revealed by antibody-mediated proximity labelling coupled to mass spectrometry
Source: eLife. 2024 May 8;13:e95718. doi: 10.7554/eLife.95718 (PMC11147508; doi:10.7554/eLife.95718)

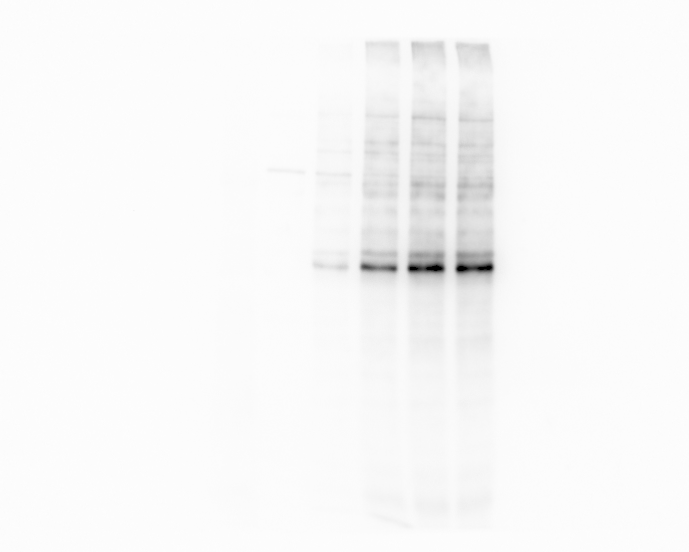

Supplement: Figure 1—figure supplement 1—source data 1. [file elife-95718-fig1-figsupp1-data1.zip › Figure 1-Supplement Figure-1/Figure 1-Supplement Figure 1(c)-source data(Chemiluminescence).raw16.tif]

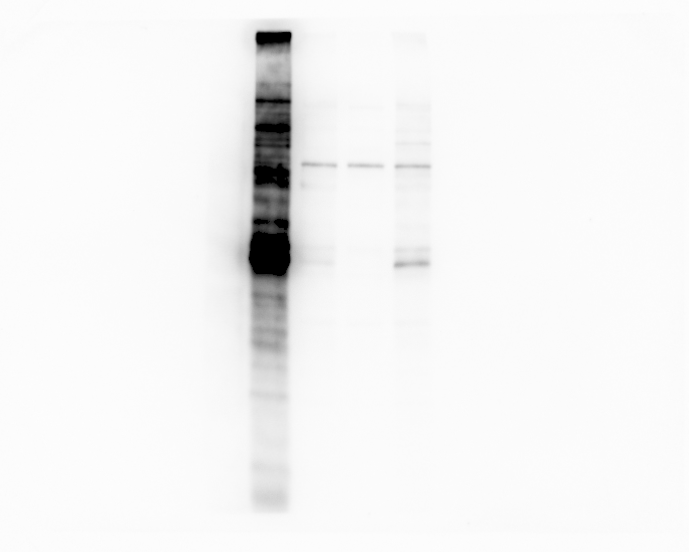

Supplement: Figure 1—figure supplement 1—source data 1. [file elife-95718-fig1-figsupp1-data1.zip › Figure 1-Supplement Figure-1/Figure 1-Supplement Figure 1(b)-source data(Chemiluminescence).raw16.tif]

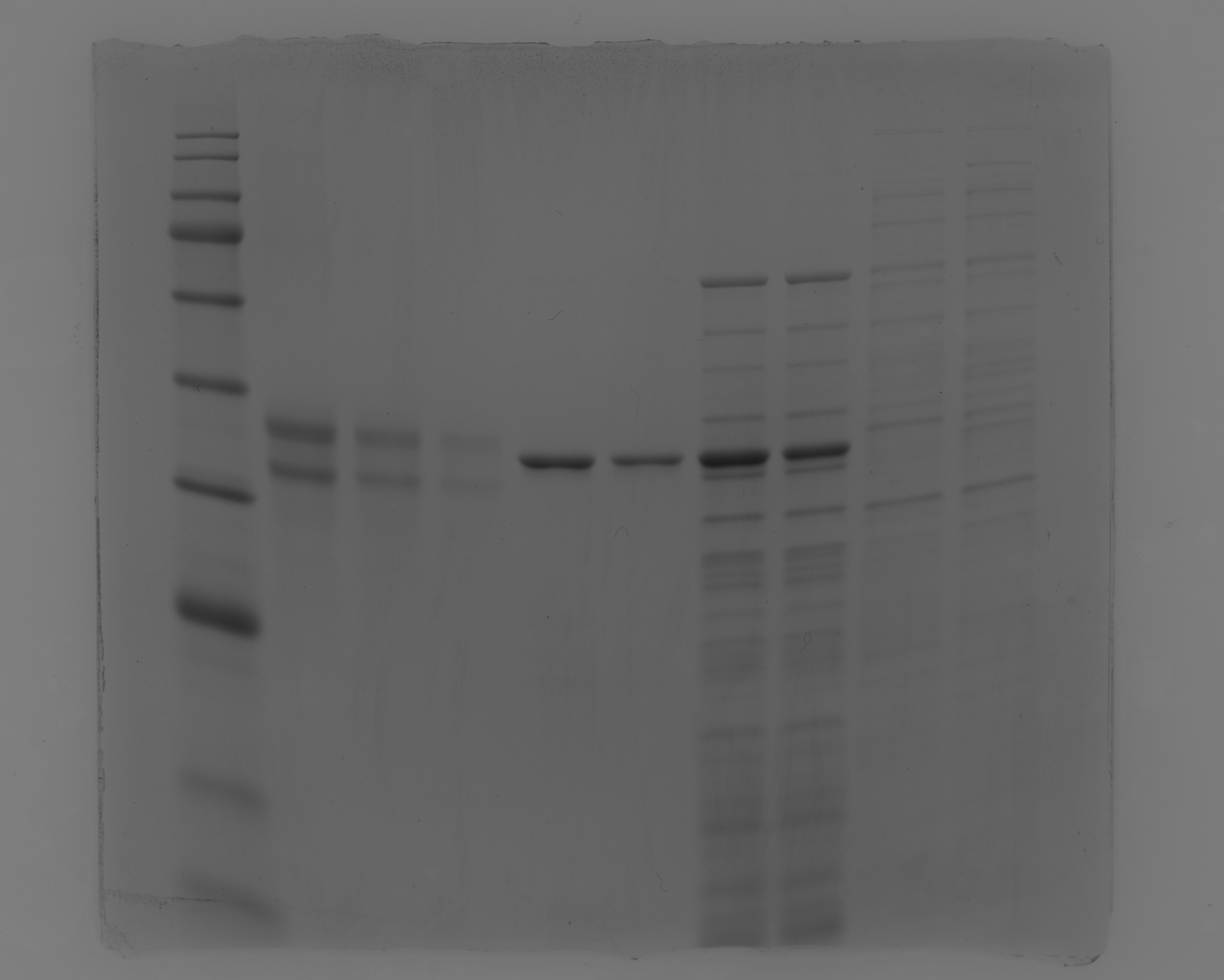

Supplement: Figure 1—figure supplement 1—source data 1. [file elife-95718-fig1-figsupp1-data1.zip › Figure 1-Supplement Figure-1/Figure 1-Supplement Figure 1(a)-source data 1.raw16.tif]

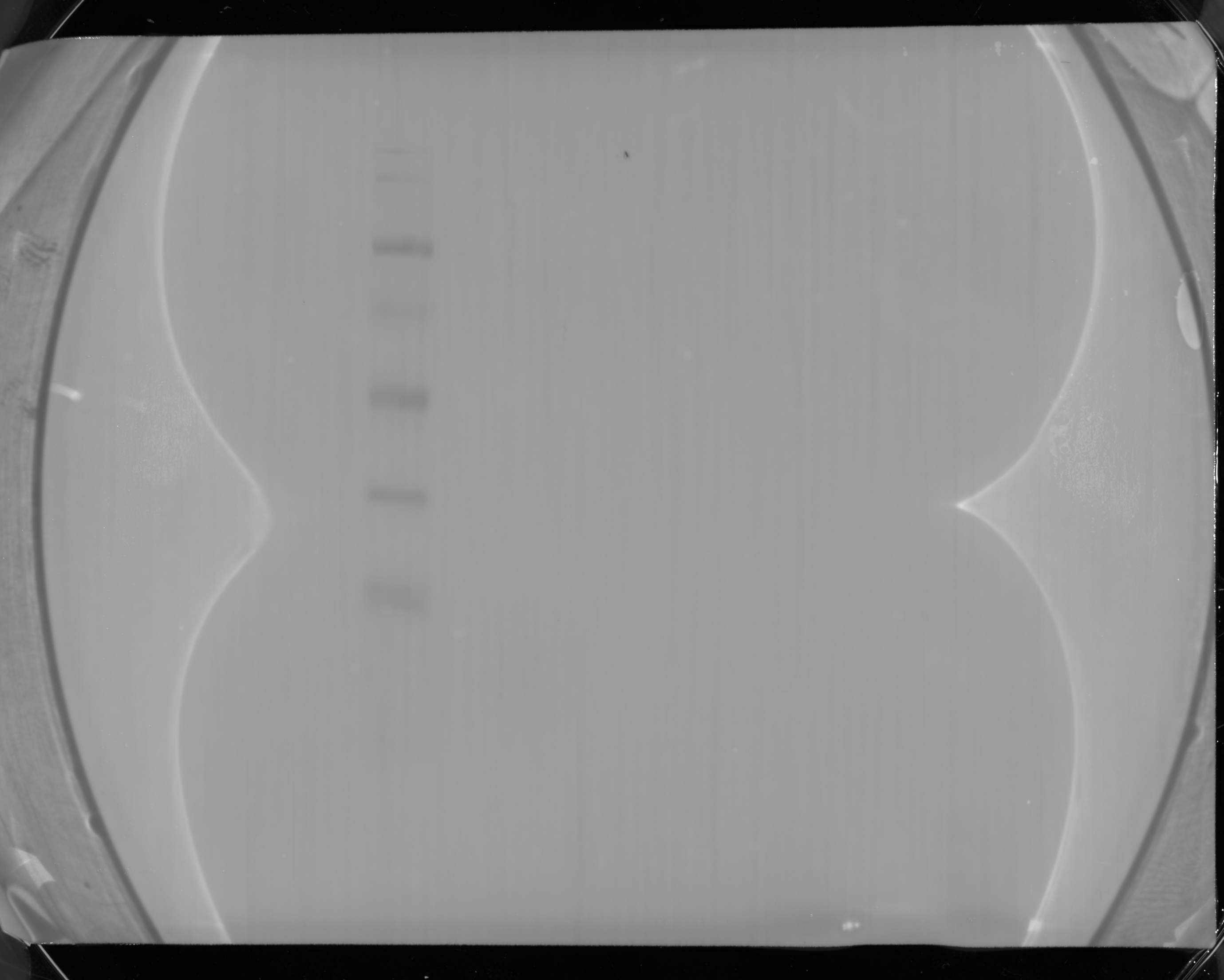

Supplement: Figure 1—figure supplement 1—source data 1. [file elife-95718-fig1-figsupp1-data1.zip › Figure 1-Supplement Figure-1/Figure 1-Supplement Figure 1(b)-source data(Colorimetric).raw16.tif]

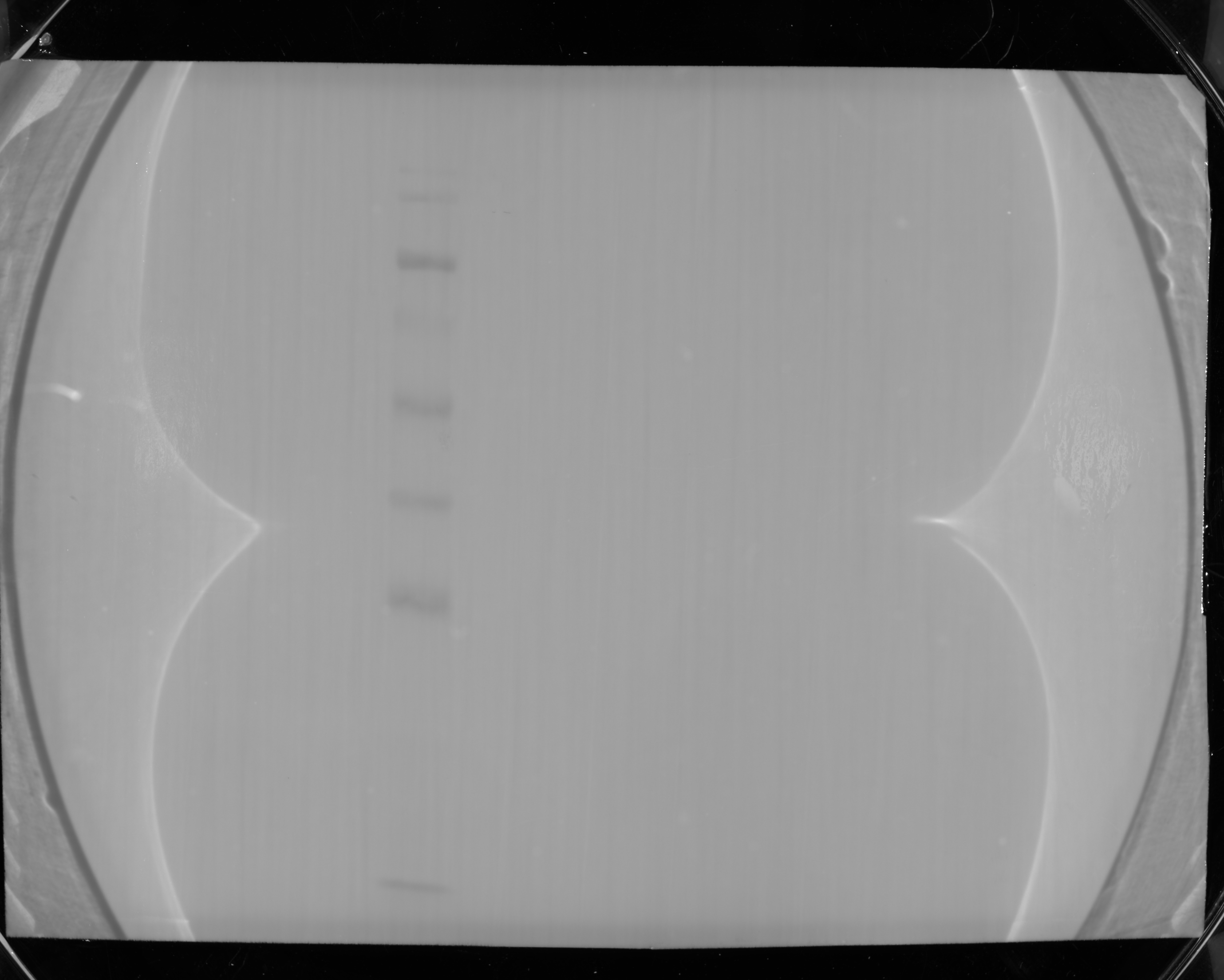

Supplement: Figure 1—figure supplement 1—source data 1. [file elife-95718-fig1-figsupp1-data1.zip › Figure 1-Supplement Figure-1/Figure 1-Supplement Figure 1(c)-source data(Colorimetric).raw16.tif]

# Figure 1-Supplement Figure 1(c)-source data (Colorimetric)

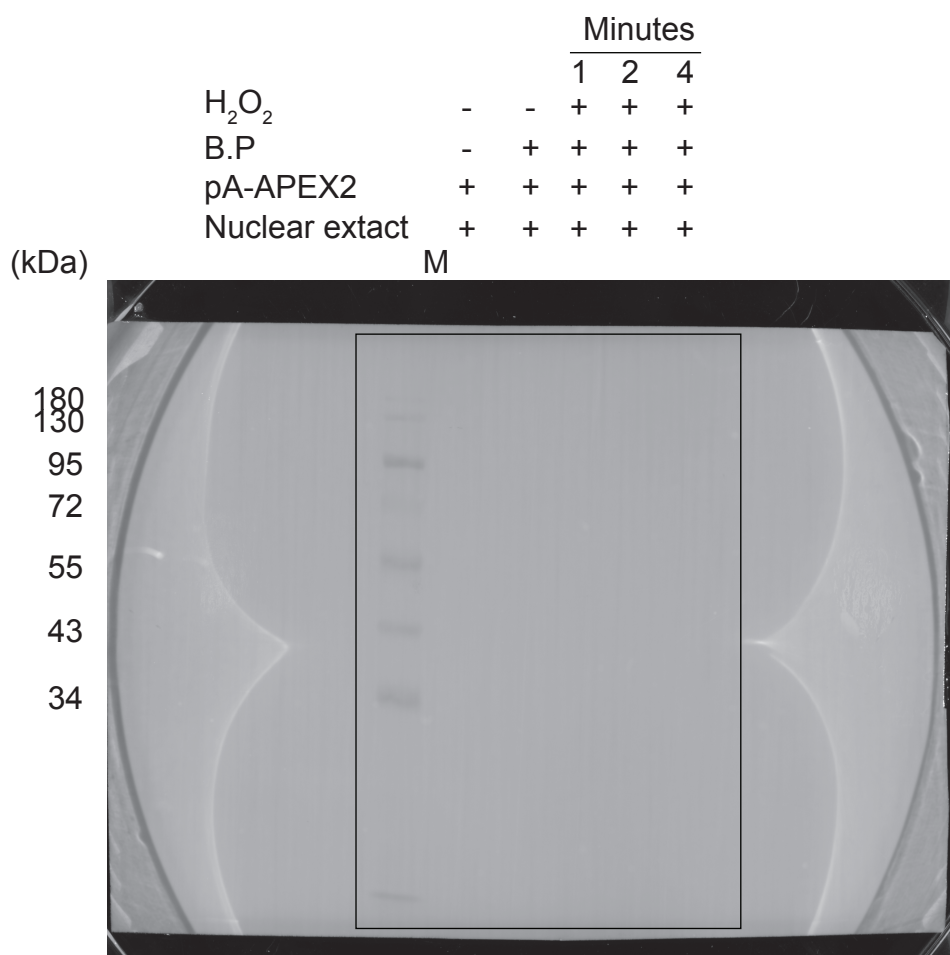

Supplement: Figure 1—figure supplement 1—source data 2. [file elife-95718-fig1-figsupp1-data2.zip › Figure 1-Supplement Figure-1-2/Figure 1-Supplement Figure 1(c)-source data(Colorimetric).raw16.pdf]

Figure 1-Supplement Figure 1(b)-source data  
(Colorimetric)

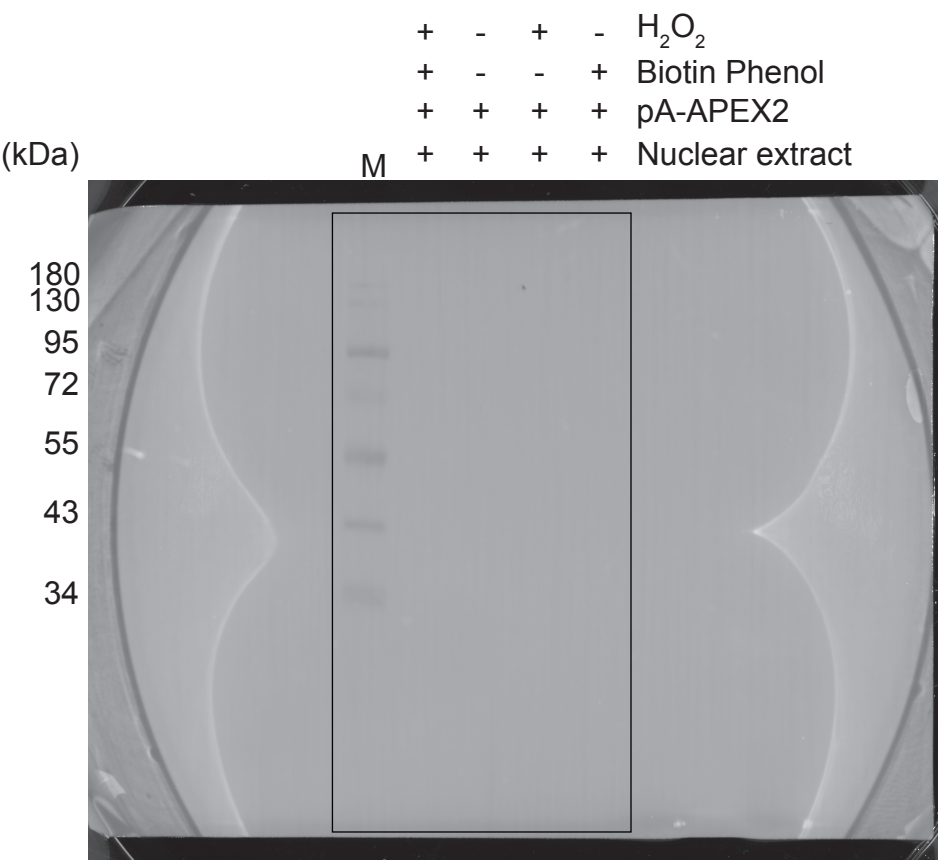

Supplement: Figure 1—figure supplement 1—source data 2. [file elife-95718-fig1-figsupp1-data2.zip › Figure 1-Supplement Figure-1-2/Figure 1-Supplement Figure 1(b)-source data(Colorimetric).raw16.pdf]

Figure 1-Supplement Figure 1(a)-source data

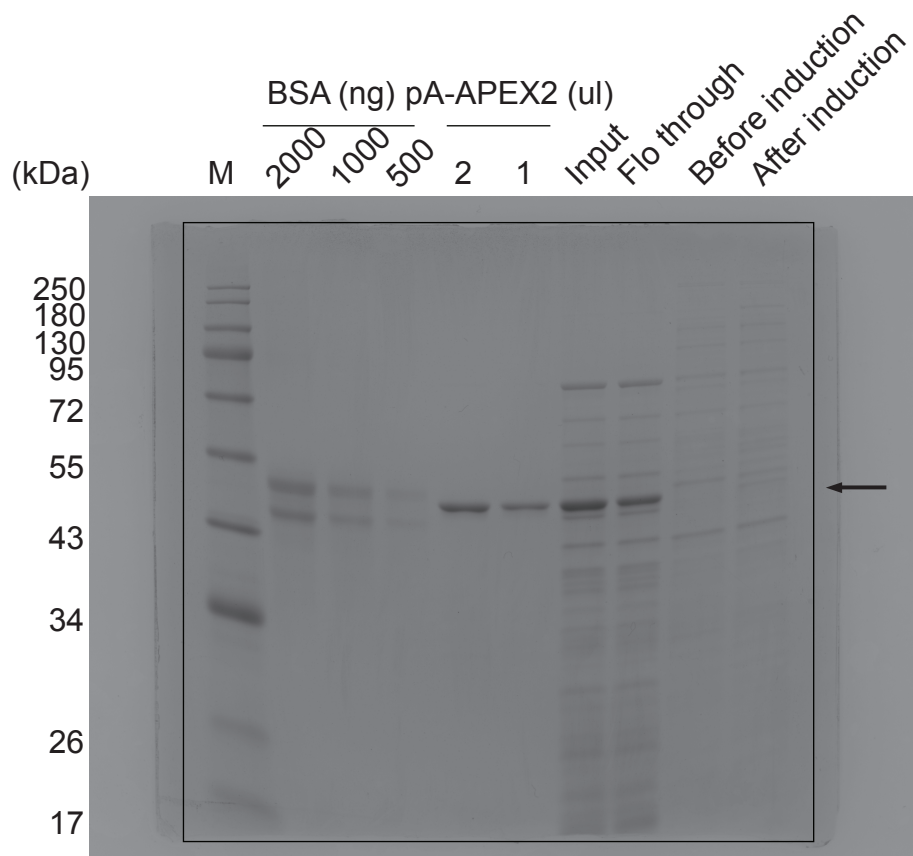

Supplement: Figure 1—figure supplement 1—source data 2. [file elife-95718-fig1-figsupp1-data2.zip › Figure 1-Supplement Figure-1-2/Figure 1-Supplement Figure 1(a)-source data 1.raw16.pdf]

Figure 1-Supplement Figure 1(b)-source data  
(Chemiluminescence)

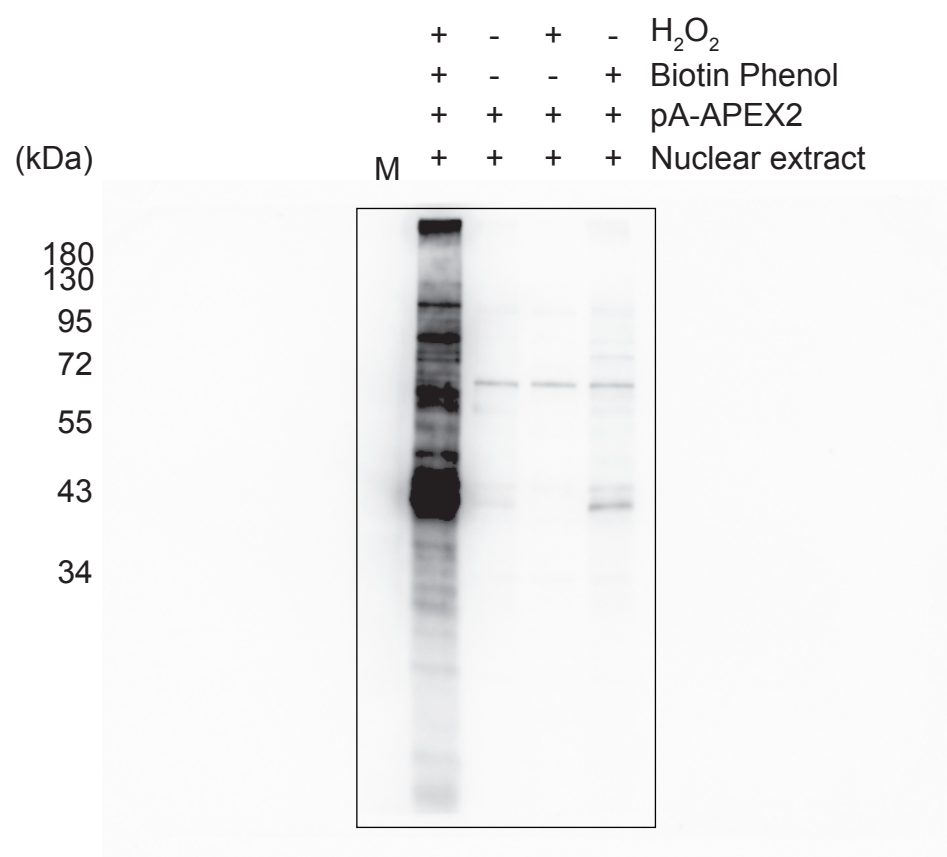

Supplement: Figure 1—figure supplement 1—source data 2. [file elife-95718-fig1-figsupp1-data2.zip › Figure 1-Supplement Figure-1-2/Figure 1-Supplement Figure 1(b)-source data(Chemiluminescence).raw16.pdf]

Figure 1-Supplement Figure 1(c)-source data  
(Chemiluminescence)

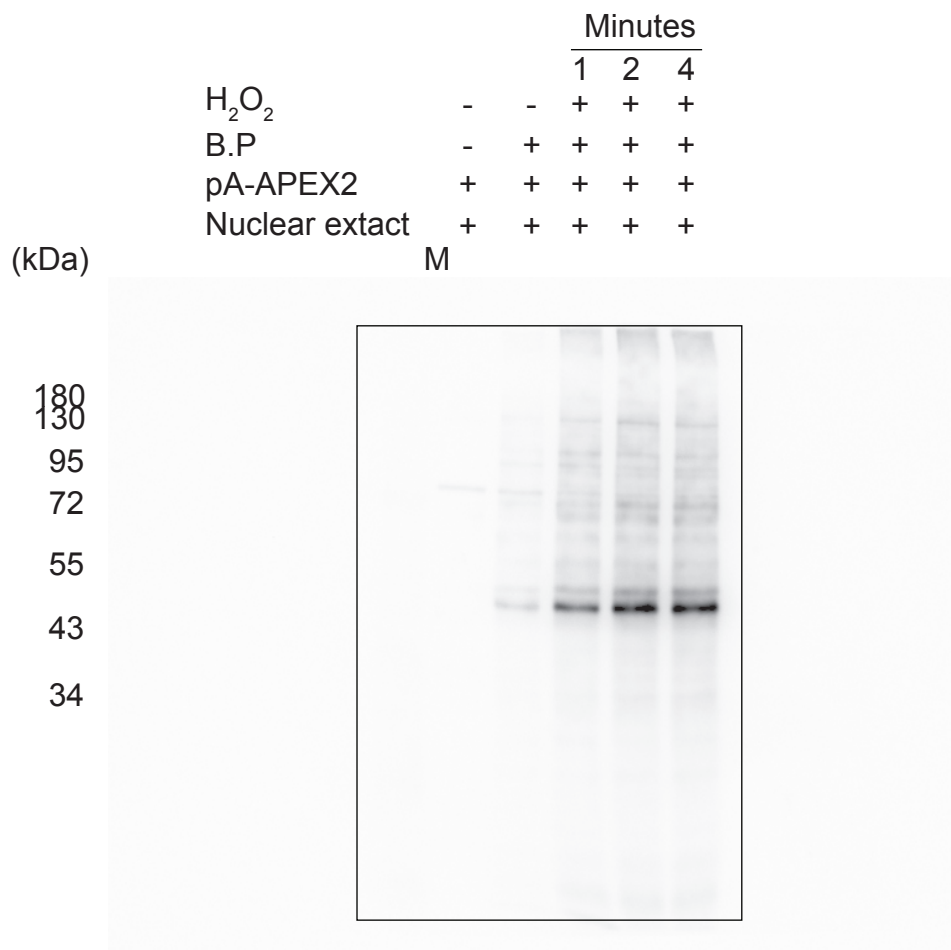

Supplement: Figure 1—figure supplement 1—source data 2. [file elife-95718-fig1-figsupp1-data2.zip › Figure 1-Supplement Figure-1-2/Figure 1-Supplement Figure 1(c)-source data(Chemiluminescence).raw16.pdf]

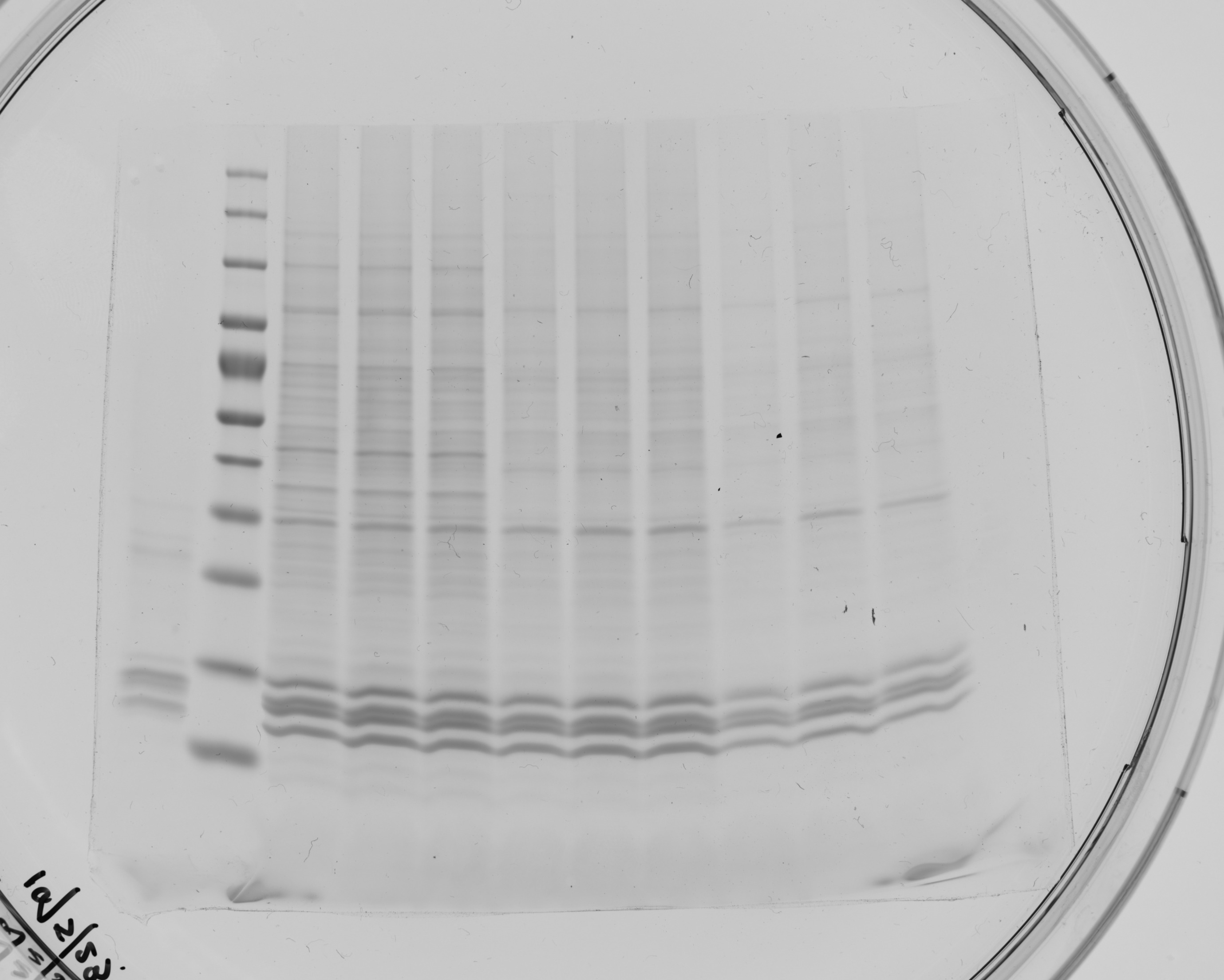

Supplement: Figure 2—figure supplement 1—source data 1. [file elife-95718-fig2-figsupp1-data1.zip › Figure 2-Supplement Figure-2/Figure 2-Supplement Figure 2(g)-source data(Coomassie Blue).raw16.tif]

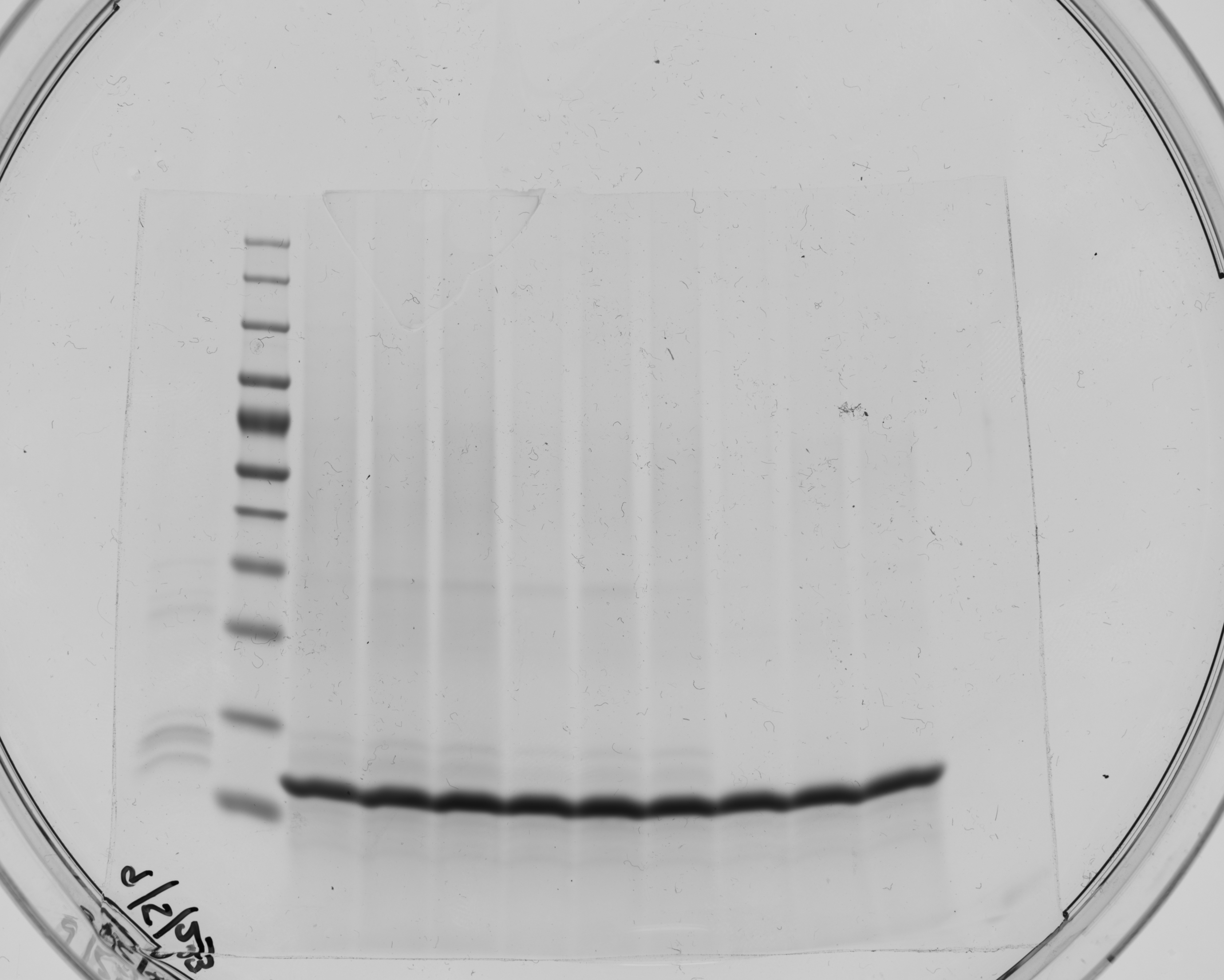

Supplement: Figure 2—figure supplement 1—source data 1. [file elife-95718-fig2-figsupp1-data1.zip › Figure 2-Supplement Figure-2/Figure 2-Supplement Figure 2(h)-source data(Coomassie Blue).raw16.tif]

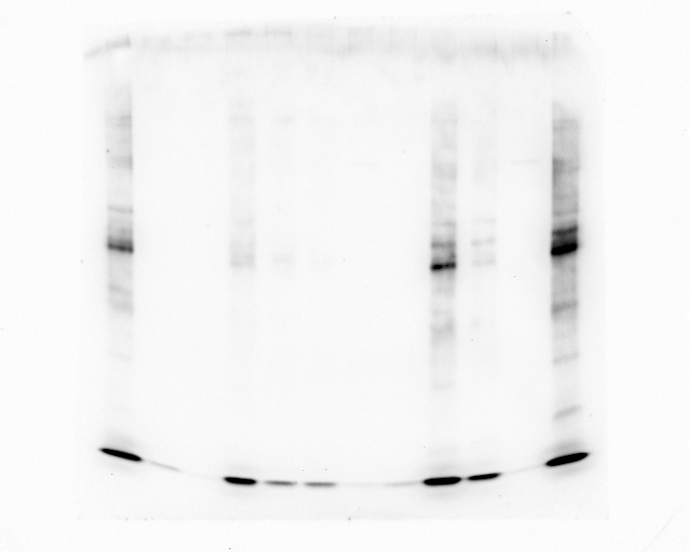

Supplement: Figure 2—figure supplement 1—source data 1. [file elife-95718-fig2-figsupp1-data1.zip › Figure 2-Supplement Figure-2/Figure 2-Supplement Figure 2(a)-source data(Chemiluminescence).raw16.tif]

Figure 2-Supplement Figure 2(h)-source data  
(Coomassie Blue)

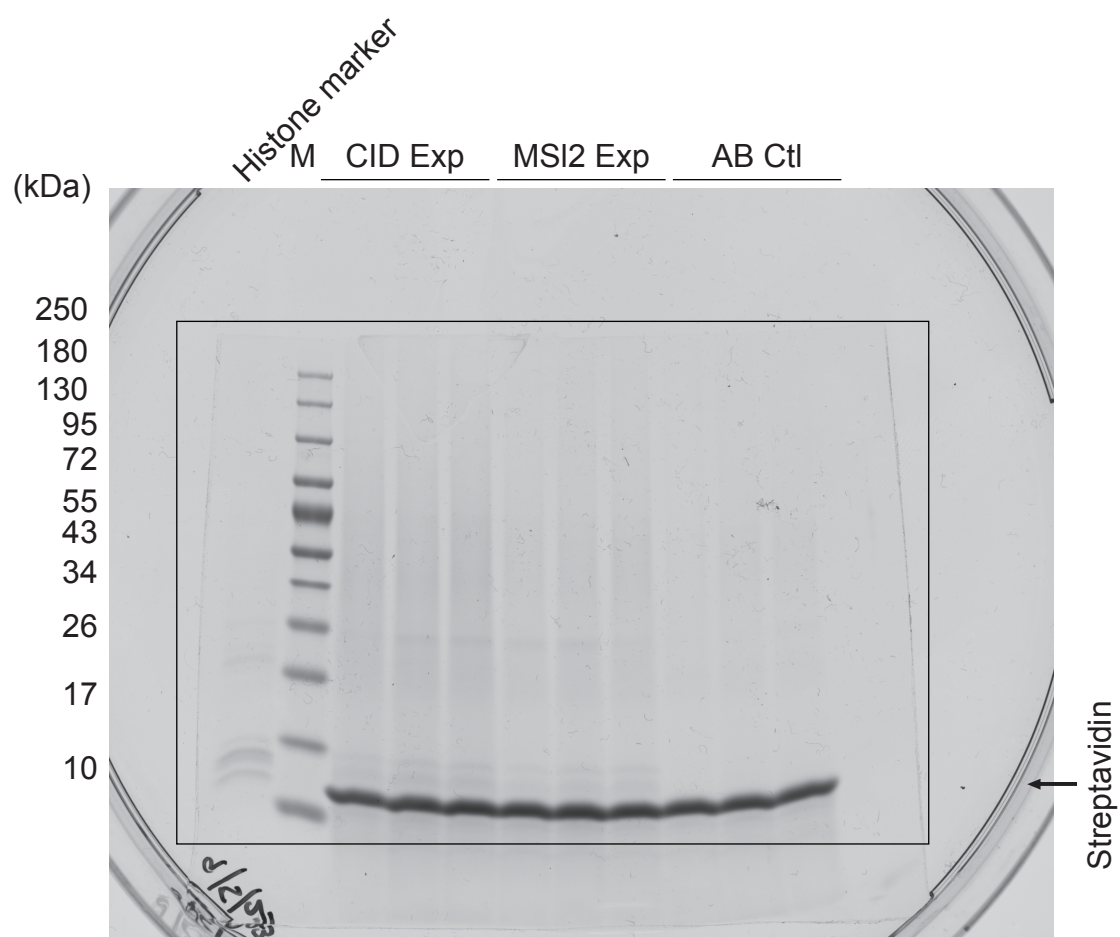

Supplement: Figure 2—figure supplement 1—source data 2. [file elife-95718-fig2-figsupp1-data2.zip › Figure 2-Supplement Figure-2/Figure 2-Supplement Figure 2(h)-source data(Coomassie Blue).raw16.pdf]

Figure 2-Supplement Figure 2(g)-source data  
(Coomassie Blue)

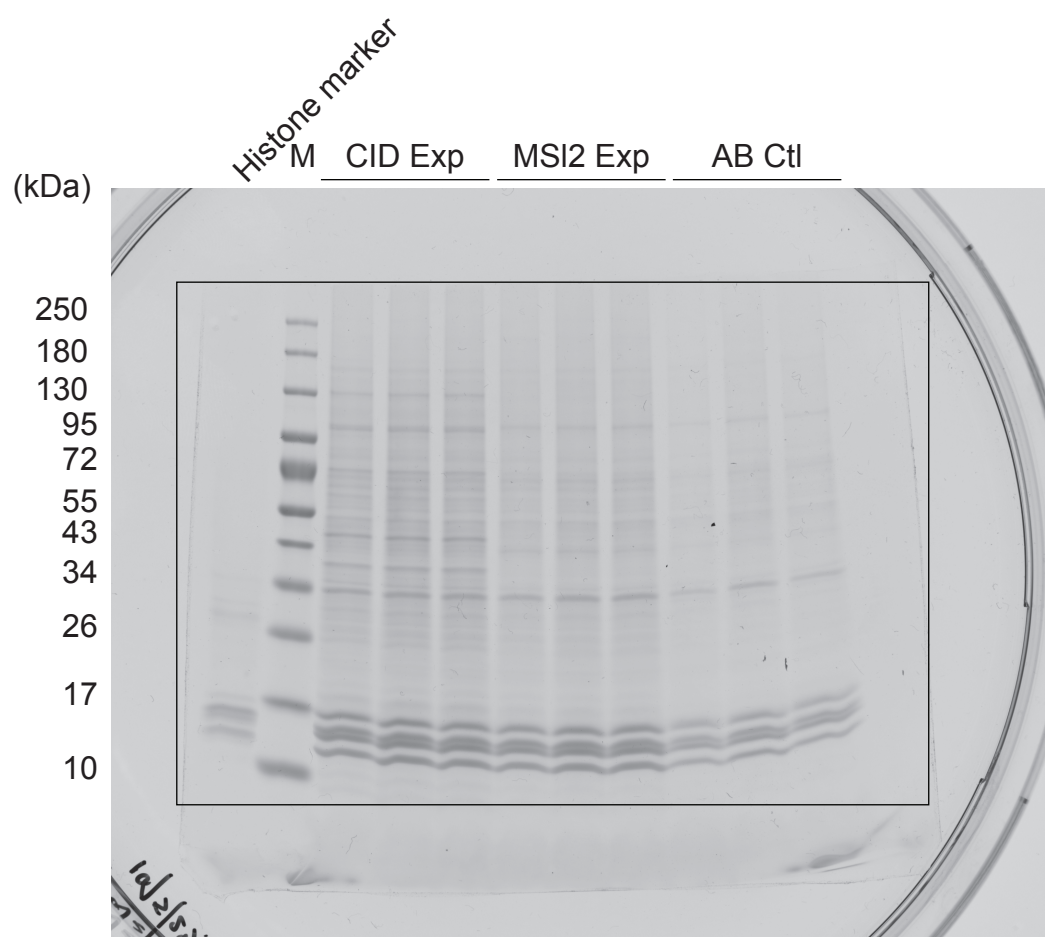

Supplement: Figure 2—figure supplement 1—source data 2. [file elife-95718-fig2-figsupp1-data2.zip › Figure 2-Supplement Figure-2/Figure 2-Supplement Figure 2(g)-source data(Coomassie Blue).raw16.pdf]

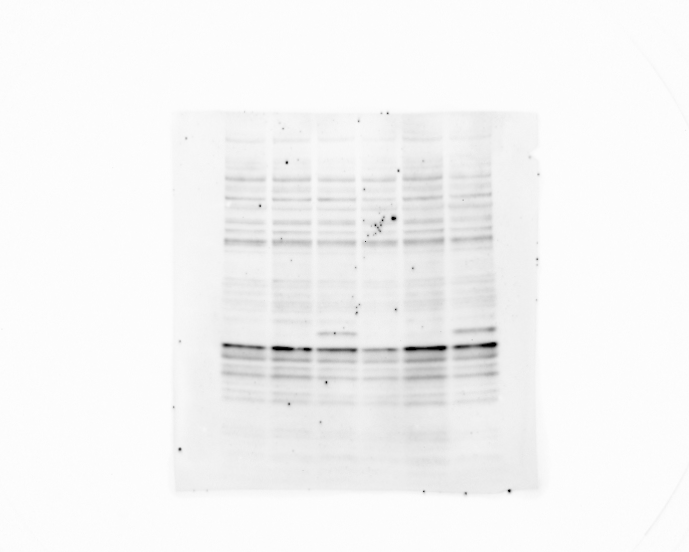

Supplement: Figure 5—figure supplement 1—source data 1. [file elife-95718-fig5-figsupp1-data1.zip › Figure 5-Supplement Figure-6/Figure 5-Supplement Figure 6(a)-source data(Chemiluminescence).raw16.tif]

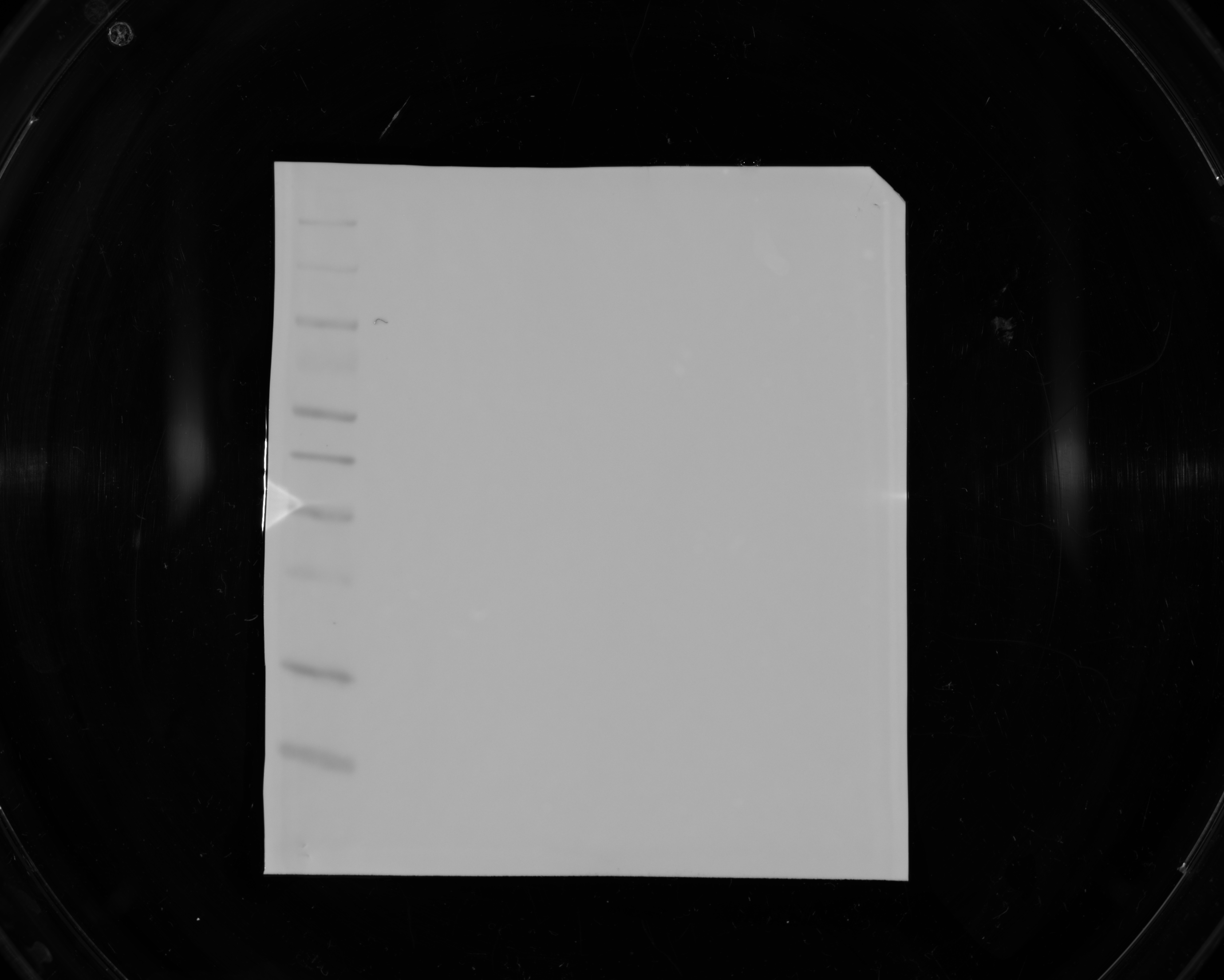

Supplement: Figure 5—figure supplement 1—source data 1. [file elife-95718-fig5-figsupp1-data1.zip › Figure 5-Supplement Figure-6/Figure 5-Supplement Figure 6(b)-source data(Colorimetric).raw16.tif]

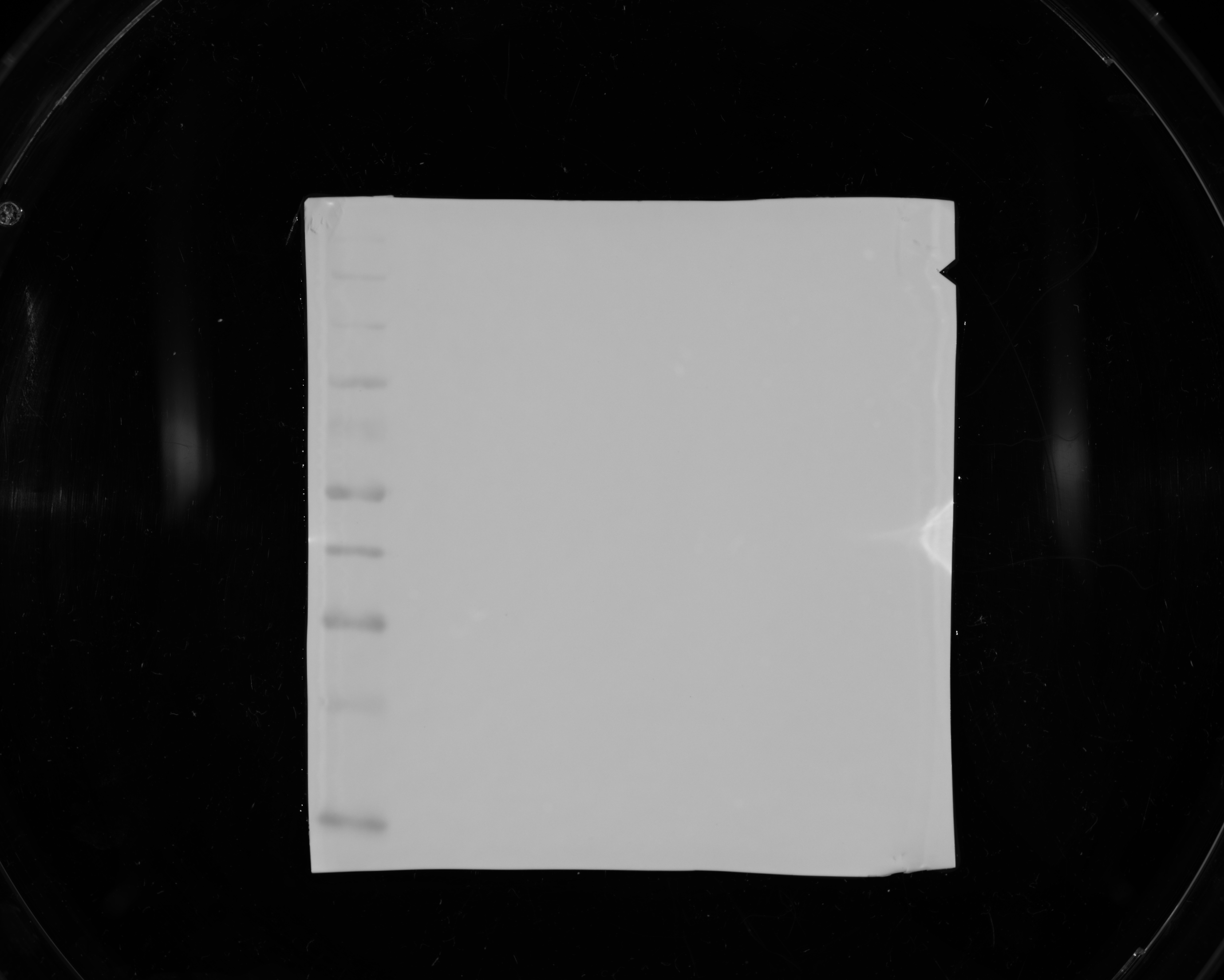

Supplement: Figure 5—figure supplement 1—source data 1. [file elife-95718-fig5-figsupp1-data1.zip › Figure 5-Supplement Figure-6/Figure 5-Supplement Figure 6(a)-source data(Colorimetric).raw16.tif]

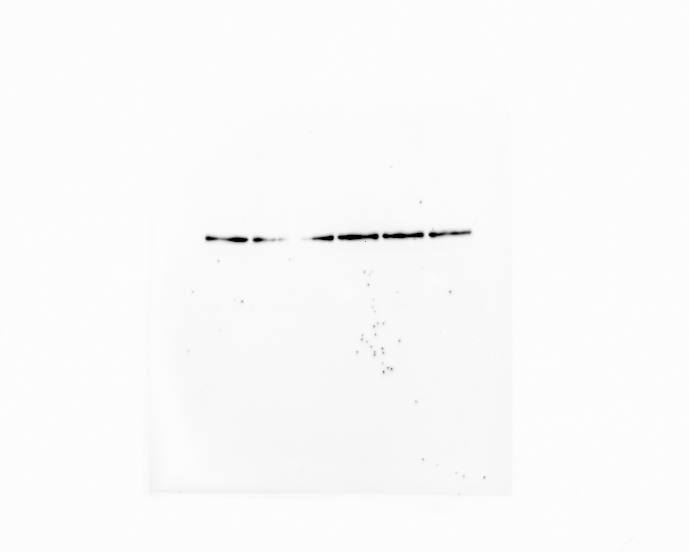

Supplement: Figure 5—figure supplement 1—source data 1. [file elife-95718-fig5-figsupp1-data1.zip › Figure 5-Supplement Figure-6/Figure 5-Supplement Figure 6(b)-source data(Chemiluminescence).raw16.tif]

Figure 5-Supplement Figure 6(b)-source data  
((Colorimetric))

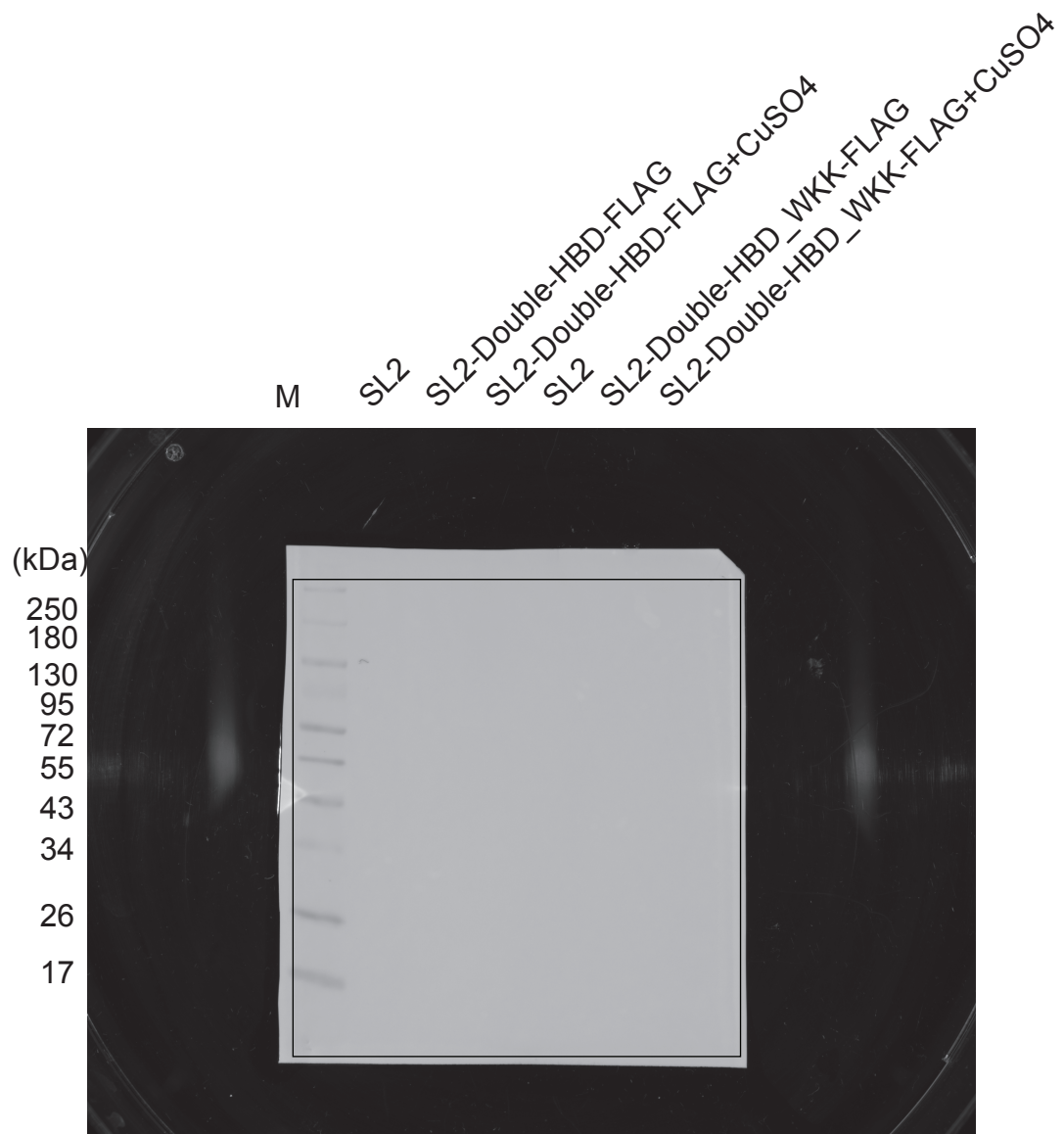

Supplement: Figure 5—figure supplement 1—source data 2. [file elife-95718-fig5-figsupp1-data2.zip › Figure 5-Supplement Figure-6/Figure 5-Supplement Figure 6(b)-source data(Colorimetric).raw16.pdf]

Figure 5-Supplement Figure 6(a)-source data  
(Chemiluminescence)

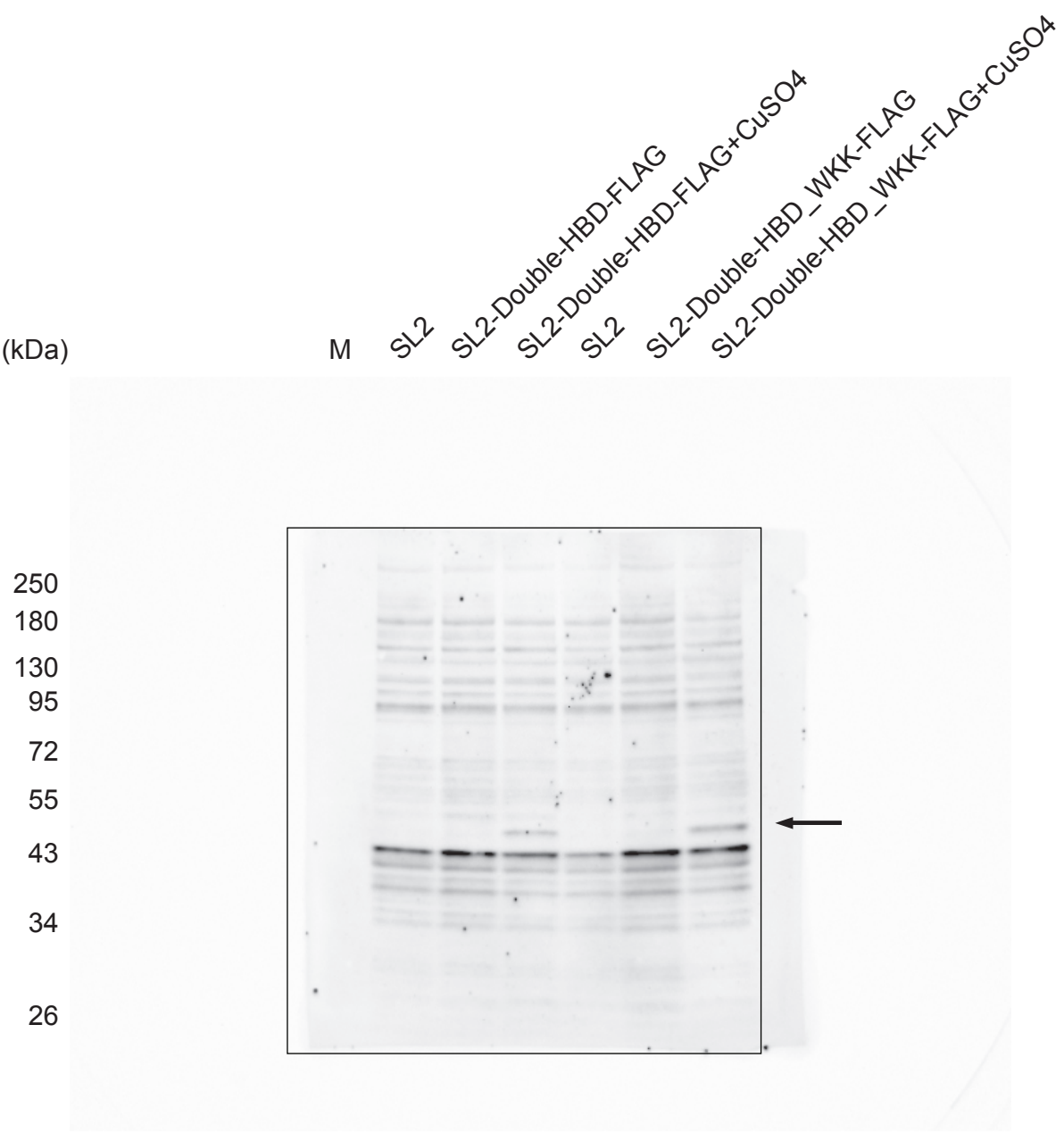

Supplement: Figure 5—figure supplement 1—source data 2. [file elife-95718-fig5-figsupp1-data2.zip › Figure 5-Supplement Figure-6/Figure 5-Supplement Figure 6(a)-source data(Chemiluminescence).raw16.pdf]

Figure 5-Supplement Figure 6(b)-source data  
(Chemiluminescence)

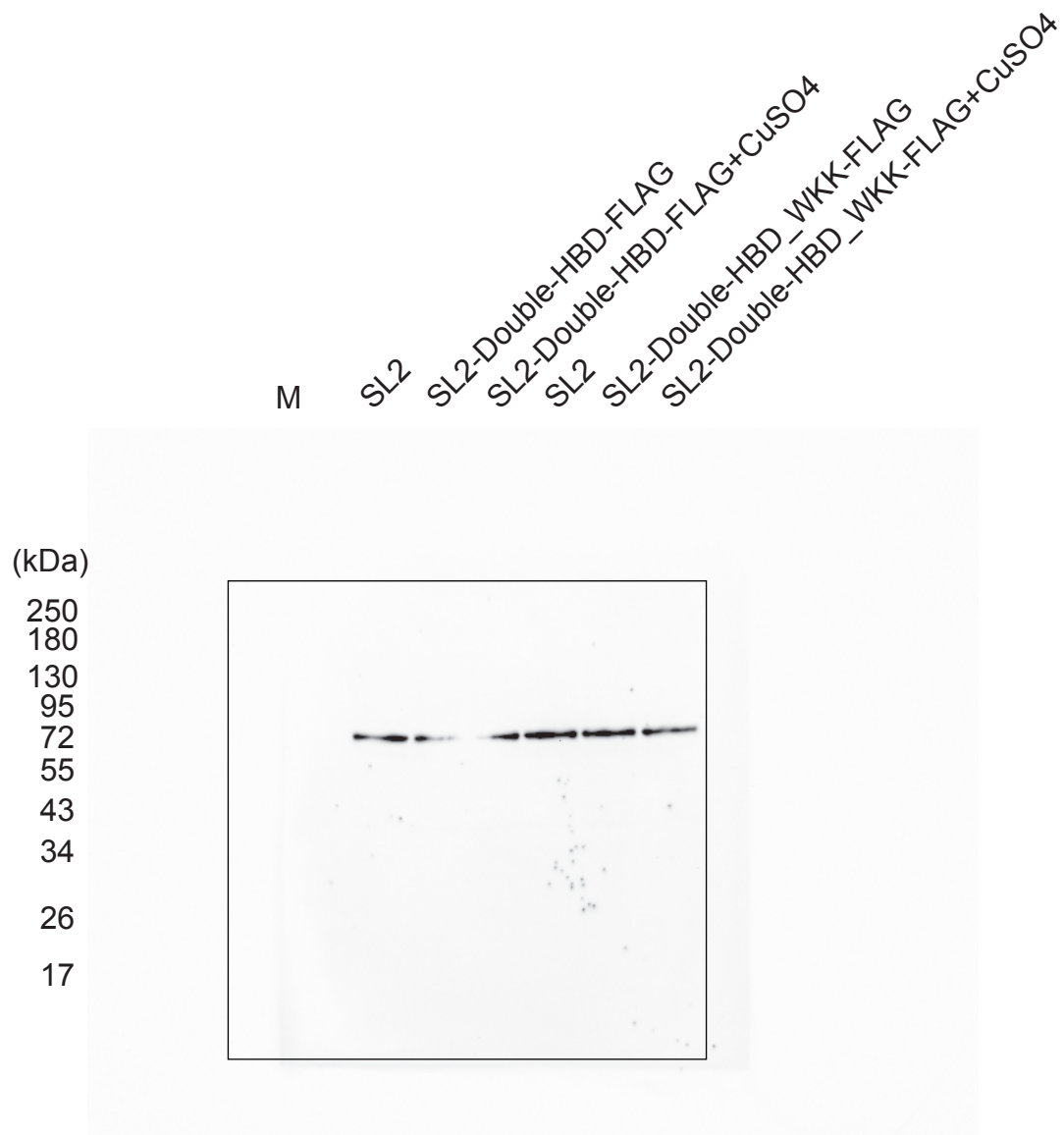

Supplement: Figure 5—figure supplement 1—source data 2. [file elife-95718-fig5-figsupp1-data2.zip › Figure 5-Supplement Figure-6/Figure 5-Supplement Figure 6(b)-source data(Chemiluminescence).raw16.pdf]

Figure 5-Supplement Figure 6(a)-source data  
(Colorimetric)

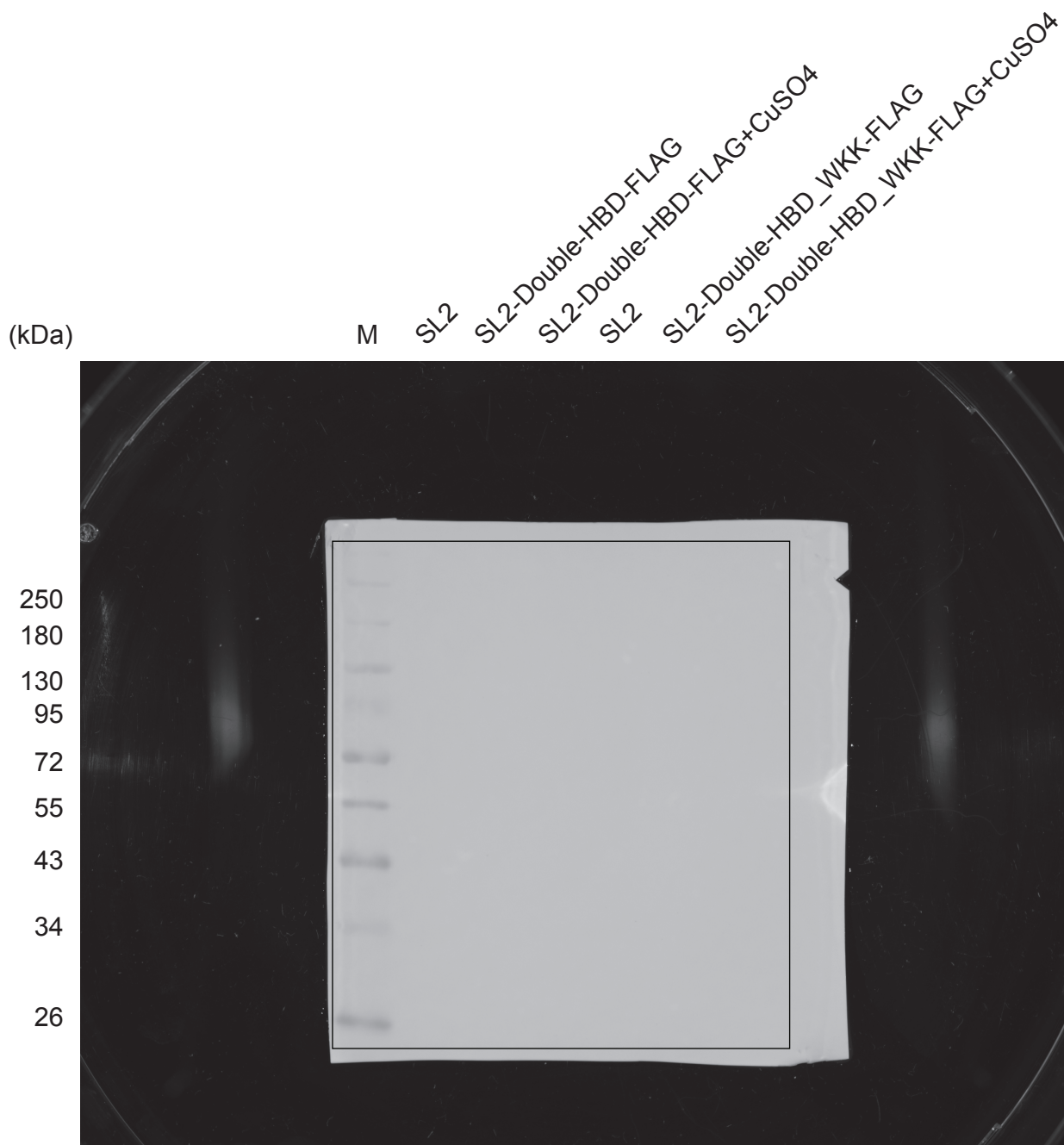

Supplement: Figure 5—figure supplement 1—source data 2. [file elife-95718-fig5-figsupp1-data2.zip › Figure 5-Supplement Figure-6/Figure 5-Supplement Figure 6(a)-source data(Colorimetric).raw16.pdf]
