# Supplementary material for: The role of RNA in the maintenance of chromatin domains as revealed by antibody-mediated proximity labelling coupled to mass spectrometry
Source: eLife. 2024 May 8;13:e95718. doi: 10.7554/eLife.95718 (PMC11147508; doi:10.7554/eLife.95718)

Figure 2-Supplement Figure 2(a)-source data (Chemiluminescence)

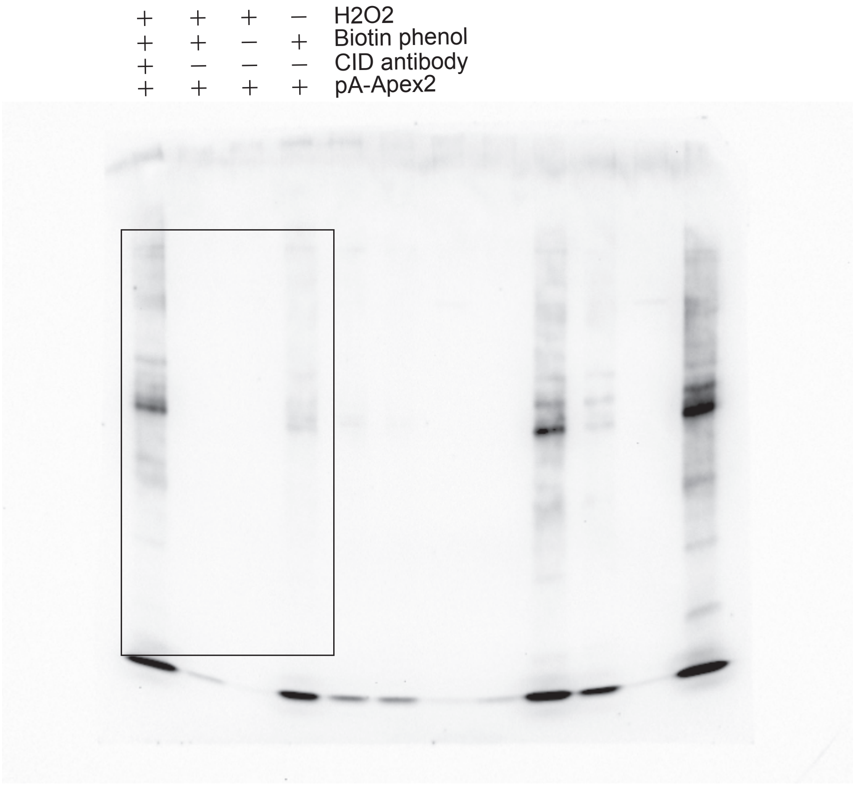

Supplement: Figure 2—figure supplement 1—source data 2. [file elife-95718-fig2-figsupp1-data2.zip › Figure 2-Supplement Figure-2/Figure 2-Supplement Figure 2(a)-source data(Chemiluminescence).raw16.pdf]
